# Krankenhausversorgung im Krisenfall: Sind deutsche Kliniken auf militärische Auseinandersetzungen vorbereitet?

**DOI:** 10.1007/s00101-026-01738-3

**Published:** 2026-09-17

**Authors:** E. G. Pfenninger, M. Königsdorfer, S. Witte, S. Mayer, U. X. Kaisers, B. Friemert

**Affiliations:** 1 Abteilung für Anästhesiologie, Intensivmedizin und Schmerztherapie, SRH-Klinikum Sigmaringen, Sigmaringen, Deutschland; 2 Stabsstelle Resilienz- und Krisenmanagement, Universitätsklinikum Ulm, Ulm, Deutschland; 3 Klinik für Anästhesiologie und Intensivmedizin, Universitätsklinikum Ulm, Ulm, Deutschland; 4 Stabsstelle Strategisches Medizincontrolling, Universitätsklinikum Ulm, Ulm, Deutschland; 5 Klinikumsvorstand, Universitätsklinikum Ulm, Ulm, Deutschland; 6 Zentrales Klinisches Prozess, Leistungs- und Resourcenmanagement, Bundeswehrkrankenhaus Ulm, Ulm, Deutschland; 7 Universitätsklinikum Ulm, Albert-Einstein-Allee 23, 89081 Ulm, Deutschland

**Keywords:** Bewaffneter Konflikt, Verteidigungsfall, Klinische Versorgung, Zivil-Militärische Zusammenarbeit, Resilienz, Armed conflict, Defence situation, Clinical care, Civil-military cooperation, Resilience

## Abstract

**Hintergrund:**

Vor dem Hintergrund veränderter sicherheitspolitischer Rahmenbedingungen in Europa stellt sich die Frage, inwieweit das deutsche Krankenhauswesen auf militärische Konfliktszenarien, Szenarien wie Katastrophe, Krise, Krieg (3K), vorbereitet ist.

**Methodik:**

Die Arbeit wurde als narrative Literaturübersicht durchgeführt und umfasste den Zeitraum Januar 2020 bis 0Juni 2026 in den Datenbanken PubMed und Google Scholar. Verwendet wurden Kombinationen der Suchbegriffe „hospital preparedness“, „hospital resilience“, „war“, „military conflict“, „mass casualty incident“, „disaster planning“, „Germany“ sowie deren deutschsprachige Entsprechungen. Die Suchbegriffe wurden sowohl einzeln als auch in verschiedenen Kombinationen eingesetzt.

**Ergebnisse:**

Kontrollierte, randomisierte und verblindete Studien (RCT) zur Frage, ob deutsche Krankenhäuser auf militärische Auseinandersetzungen vorbereitet sind, gibt es nicht. Die verfügbare Evidenz zum Thema besteht überwiegend aus Expertenempfehlungen, Behördenberichten und Erfahrungen aus aktuellen militärischen Konflikten.

**Diskussion und Schlussfolgerung:**

Während Deutschland über ein hochentwickeltes ziviles Gesundheitssystem verfügt, zeigen aktuelle Studien und nationale Risikoanalysen relevante Defizite in der Vorbereitung auf großflächige militärische Konflikte, insbesondere hinsichtlich eines lang andauernden Massenanfalls von Verletzten und der generellen systemischen Resilienz der Gesundheitsversorgung. Die Erfahrungen aus dem Russisch-Ukrainischen Krieg verdeutlichen, dass moderne Kriege zu einer massiven Belastung der Gesundheitssysteme führen. Die Integration ziviler Krankenhäuser in die nationale Sicherheitsplanung sowie regelmäßige Übungen und Investitionen in Resilienz tragen entscheidend dazu bei, die medizinische Versorgung auch in 3K-Situationen aufrechtzuerhalten.

## Abstract (en)

**Background:**

Considering the evolving security policy framework conditions in Europe, it is imperative to assess if the German hospital system is prepared to face military conflict scenarios, such as disasters, crises and wars.

**Methods:**

This study was conducted as a narrative literature review and covered the period from January 2020 to June 2026 in the PubMed and Google Scholar databases. The study used combinations of the search terms “hospital preparedness”, “hospital resilience”, “war”, “military conflict”, “mass casualty incident”, “disaster planning”, “Germany” and their German language equivalents. The search terms were used both individually and in various combinations. The study included original articles, reviews, guidelines, government reports and health policy publications in both German and English that addressed hospital preparedness for armed conflicts or comparable large-scale disasters. Publications without a direct connection to hospital care in crisis situations as well as those for which the full text was not available, were excluded.

**Results:**

There are no controlled, randomized and blinded studies (RCT) examining the question whether German hospitals are prepared for military conflicts. The available evidence on this topic consists primarily of expert recommendations, government reports and experiences from current military conflicts.

**Discussion and conclusion:**

Germany has a highly developed civilian healthcare system but recent studies and official risk analyses reveal shortcomings in its preparedness for large-scale military conflicts. There are particular concerns about its ability to handle long-lasting mass casualty incidents (MCI), and the systemic resilience of the healthcare system in general. Experience from the Russia-Ukraine war shows that modern wars generate enormous numbers of seriously injured patients and quickly push the healthcare system to its limits. While smaller influxes of wounded would be manageable, a large-scale military conflict would require additional military, logistical and international support. Challenges lie not only in infrastructure, but also in the availability of qualified personnel and intensive care capacity as well as in logistical resilience. Greater integration of civilian hospitals into national security planning, along with regular exercises and investments in resilience, could be the key to ensuring medical care during a military crisis.

## Einleitung

Die sicherheitspolitische Lage Europas und Deutschlands hat sich seit dem Beginn der russischen Invasion in der Ukraine im Februar 2022 tiefgreifend verändert. Lange durch das Selbstverständnis als „friedenssichernde“, defensiv ausgerichtete Nation geprägt, führt die Bundesrepublik heute eine Debatte über konkrete militärische Bedrohungen, Verteidigungsbereitschaft und die zukünftige Rolle innerhalb der NATO [1]. Es geht um die Frage, wie Deutschland auf Katastrophen, Krisen und Krieg (3K) vorbereitet ist, wobei die Frage eines militärischen Konfliktes (Bündnis- und Landesverteidigung) im Fokus steht. Deutschland würde aufgrund seiner geostrategischen Lage und seiner leistungsfähigen zivilen Gesundheitsinfrastruktur eine zentrale Rolle bei der Versorgung von Verwundeten, der Behandlung ziviler Opfer sowie der Aufrechterhaltung der allgemeinen Gesundheitsversorgung spielen [2]. Dies bedeutet, unabhängig davon, wie leistungsfähig unser Gesundheitssystem ist, müssen die eigene Bevölkerung, verwundete deutsche Soldaten sowie verwundete NATO-Soldaten versorgt werden. Neben militärischen Fähigkeiten rückt zunehmend die zivile Resilienz – unter anderem das Gesundheitswesen – in den Fokus, wobei laut der Weltgesundheitsorganisation (WHO) Resilienz von Gesundheitssystemen die Fähigkeit bedeutet, Krisen zu bewältigen, wichtige Funktionen aufrechtzuerhalten und sich anzupassen oder zu transformieren, um nach einer Störung gestärkt weiter zu bestehen [3]. In Artikel 3 „Resilience, civil preparedness“ der NATO heißt es: „Each NATO member country needs to be resilient in order to withstand a major shock such as … armed attack“ [4]. „Civil preparedness“ betrifft nicht allein militärische Sanitätsstrukturen, sondern vor allem die quantitative und qualitative Leistungsfähigkeit der zivilen Krankenhäuser bei massiver und langanhaltender Belastung [5], da die die Bundeswehr selbst nicht in der Lage ist, die verwundeten Soldaten in ihren Bundeswehrkrankenhäusern zu versorgen. Gleichzeitig werden zivile medizinische Einrichtungen zunehmend selbst zu Angriffszielen, wodurch die Funktionsfähigkeiten der medizinischen Infrastruktur gefährdet und reduziert werden [6].

Die Anästhesiologie spielt aufgrund ihrer zentralen Funktion in der präklinischen Versorgung, der perioperativen Stabilisierung sowie der intensivmedizinischen Weiterbehandlung eine Schlüsselrolle in der Versorgung schwer verletzter Patienten und damit im Krankenhauswesen [7]. Darüber hinaus kommt der Anästhesiologie eine zentrale Rolle in der Versorgung von Kriegs- und Kampfverletzten, insbesondere im Rahmen der Atemwegsicherung, hämodynamischen Stabilisierung sowie der perioperativen und intensivmedizinischen Behandlung im Kontext kriegerischer Auseinandersetzungen zu [8]. Die enge Verzahnung von Anästhesie, Intensivmedizin und Notfallmedizin führt dazu, dass Anästhesiologinnen und Anästhesiologen bei kriegerischen Auseinandersetzungen zudem häufig eine koordinierende Funktion in der Versorgung Schwerverletzter übernehmen und deshalb sowohl in Kliniken als auch in unmittelbaren Gefahrenzonen zum Einsatz kommen [8].

Das Bundesamt für Bevölkerungsschutz und Katastrophenhilfe (BBK) weist darauf hin, dass der medizinische Bedarf im Verteidigungsfall deutlich steigen wird, während gleichzeitig Energie‑, IT- und Lieferketteninfrastrukturen gestört sein werden. Der gesundheitliche Bevölkerungsschutz müsse daher sowohl Kapazitätserweiterungen als auch Ressourcensicherung berücksichtigen [9, 10]. So sind im Russisch-Ukrainischen Krieg seit Februar 2022 und Dezember 2025 auf ukrainischer Seite schätzungsweise zwischen 500.000 und 600.000 Verluste, darunter Tote, Verwundete und Vermisste, und zwischen 100.000 und 140.000 Todesopfer zu verzeichnen [11]. Auf russischer Seite werden seit Februar 2022 etwa 1,2 Mio. Verluste (Getötete, Verwundete und Vermisste) und bis zu 325.000 Getötete verzeichnet [11]. Aber nicht nur europäische Konflikte beeinflussen die deutsche Sicherheitslage: Die jüngsten militärischen Auseinandersetzungen im Nahen Osten – insbesondere der Krieg zwischen den USA, Israel und dem Iran – haben den deutschen Behörden zufolge zu einer erhöhten Alarmbereitschaft geführt. Es wird vor möglichen Angriffen auf israelische oder amerikanische Einrichtungen sowie vor Cyberangriffen in Deutschland gewarnt, die aus dem Nahen Osten nach Europa zurückwirken könnten [12]. In diesem Kontext besteht für Europa eine erhebliche Herausforderung, die auf die Frage reduziert werden kann, ob Deutschland in der Lage ist, diesen Entwicklungen mit abgestimmten zivilen und militärischen Strukturen zu begegnen. 

## Material und Methode

Die Arbeit wurde als narrative Literaturübersicht durchgeführt. Ziel war es, den aktuellen Kenntnisstand zur Vorbereitung deutscher Krankenhäuser auf militärische Auseinandersetzungen und krisenbedingte Massenanfallssituationen darzustellen und kritisch einzuordnen.

Die Literaturrecherche umfasste den Zeitraum Januar 2020 bis Juni 2026 in den Datenbanken PubMed und Google Scholar. Ergänzend wurden Veröffentlichungen und Handlungsempfehlungen von Behörden und Fachgesellschaften berücksichtigt, darunter Dokumente des Bundesministeriums für Gesundheit, des Bundesamtes für Bevölkerungsschutz und Katastrophenhilfe (BBK), der Deutschen Krankenhausgesellschaft sowie einschlägige nationale und internationale Leitlinien. Der Beginn der Literatursuche Januar 2020 wurde gewählt, da Deutschland sich Anfang 2020 in einer Phase relativer sicherheitspolitischer Stabilität befand. Die Bundesregierung ging 2020 weiterhin davon aus, dass eine direkte militärische Bedrohung des NATO-Gebiets unwahrscheinlich sei [13].

Verwendet wurden Kombinationen der Suchbegriffe „hospital preparedness“, „hospital resilience“, „war“, „military conflict“, „mass casualty incident“, „disaster planning“, „Germany“ sowie deren deutschsprachige Entsprechungen. Die Suchbegriffe wurden sowohl einzeln als auch in verschiedenen Kombinationen eingesetzt.

**Abb. 1:**
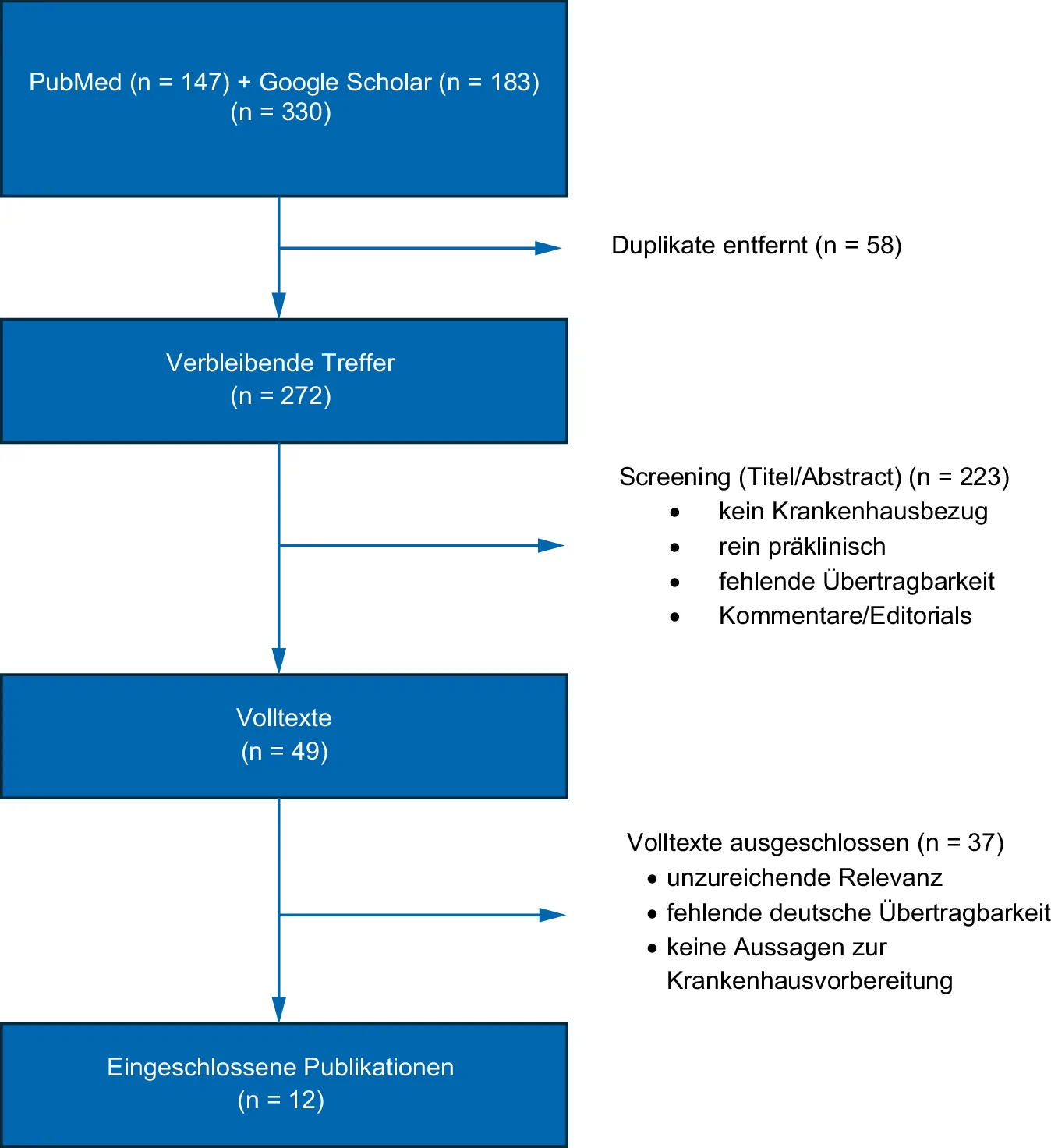
Identifikation der Literaturstellen in PubMed und Google Scholar. Suchzeitraum 01.01.2020–30.06.2026

Eingeschlossen wurden deutsch- und englischsprachige Originalarbeiten, Übersichtsarbeiten, Leitlinien, behördliche Berichte sowie gesundheitspolitische Publikationen, die einen Bezug zur Vorbereitung von Krankenhäusern auf bewaffnete Konflikte oder vergleichbare Großschadensereignisse aufwiesen. Ausgeschlossen wurden Publikationen ohne direkten Bezug zur Krankenhausversorgung im Krisenfall sowie Arbeiten, deren Volltext nicht verfügbar war. Ausgeschlossen wurden auch Publikationen zu Cyberangriffen, Hybrider Kriegsführung und zivilen Massenanfällen von Verletzten, da dies den Rahmen der Publikation sprengen würde. Die identifizierten Publikationen wurden hinsichtlich ihrer Relevanz für die Fragestellung ausgewählt (Abb. 1).

## Ergebnisse

Kontrollierte, randomisierte und verblindete Studien (RCT) zur Frage, ob deutsche Krankenhäuser auf militärische Auseinandersetzungen vorbereitet sind, gibt es nicht. Die verfügbare Evidenz zum Thema besteht überwiegend aus Expertenempfehlungen, Behördenberichten und Erfahrungen aus aktuellen Konflikten – beispielsweise dem Russisch-Ukrainischen Krieg; international vergleichende Studien sind nicht vorhanden (Tab. 1). Es werden deshalb im Folgenden weitere, auch nicht unmittelbar auf Deutschland übertragbare, Publikationen herangezogen und diskutiert.

**Tab. 1:** Eingeschlossene Studien

Autor	Jahr	Design	Setting	Wesentliche Ergebnisse	Evidenz
Wurmb et al. [18]	2024	Scoping Review	International	Eine der bislang umfassendsten Übersichtsarbeiten zur Anpassung medizinischer Standards in Katastrophen, Krisen und Kriegen. Die Autoren fordern die Entwicklung gemeinsamer Definitionen, standardisierter Krisenkonzepte und rechtssicherer Verfahren zur Ressourcenallokation, um Gesundheitssysteme besser auf zukünftige Großschadenslagen und bewaffnete Konflikte vorzubereiten	II
Thies et al. [20]	2021	Querschnittstudie (Fragebogen)	Bayern	Defizite in Ausstattung, Training und Spezialverfahren für MANV- und Terrorlagen in Krankenhäusern	III
Suda et al. [43]	2025	Systematisches Review	International	Wegen unzureichender Evidenz konnten lediglich Empfehlungen zur Schulung zur Verbesserung der Triage-Qualität sowie zu regelmäßigen Übungen zur Überprüfung des Notfallplans eines Krankenhauses ausgesprochen werden	II
Franke et al. [31]	2020	Konsensuspapier	Deutschland	Erhöhter Blutproduktbedarf bei Terrorlagen	IV
Pfenninger et al. [14]	2021	Erfahrungsbericht	Baden-Württemberg	Um Krankenhauskapazitäten nicht zu überfordern, sollte frühzeitig ein landesweites Verlegungskonzept für COVID-19-Intensivpatienten installiert werden	IV
Heller et al. [17]	2026	Modellierungsstudie	Deutschland	Zivile Kliniken müssen im Kontext der Bündnis- und Landesverteidigung die Rolle von Einrichtungen der militärischen Behandlungsebene übernehmen	III
Seyghalani et al. [44]	2024	Modellierungsstudie	International	Organisationsbezogene Resilienz ist entscheidend für die Systemstabilität von Kliniken in Krisen	III
Petanidis et al. [41]	2025	Systematisches Review	International	Effektive Krankenhauskrisenbewältigung kann nur durch die Integration von Kapazitätsplanung, KI-gestützter Prognostik und ethisch fundierten Entscheidungsmodellen erreicht werden	II
Singh et al. [5]	2026	Scoping Review	International	Die Katastrophenvorsorge in Krankenhäusern sollte als ein sich weiterentwickelnder, fortlaufender Prozess betrachtet werden. Alle Aspekte der Vorsorge sollten abdeckt werden, ebenso regionsspezifische Leitlinien, die auf die besonderen Bedürfnisse und Risiken des jeweiligen Krankenhauses zugeschnitten sind, sowie kontextbezogene Maßnahmen zur Stärkung der Widerstandsfähigkeit des Krankenhauses	II
Vulakh et al. [23]	2025	Perspektivarbeit	International	Erfahrungen aus Kriegsmedizin sind essenziell für die zivile Krankenhausvorbereitung	IV
WHO Ukraine [36]	2025	Praxisbericht	Ukraine	Seit Beginn des Krieges waren Einrichtungen der primären Gesundheitsversorgung von 39 % aller Angriffe betroffen, Einrichtungen der sekundären Gesundheitsversorgung (einschließlich Krankenhäuser) von weiteren 30 % und der Rettungsdienst (Transport) von 21 %	IV
National Academies [10]	2025	Expertenbericht	USA	Zukünftige Strategien müssen Energie‑, Wasser‑, IT-, Lieferketten- und Personalresilienz gemeinsam betrachten, um die Kontinuität der Gesundheitsversorgung in Krisen sicherzustellen	IV

Evidenzlevel: Level II: Review; Level III: Modellierung, Beobachtungsstudien; Level IV: Expertenkonsens (Oxford Centre for Evidence-Based Medicine (OCEBM) Levels of Evidence).

## Diskussion

### Ist-Zustand

Deutschland verfügt im internationalen Vergleich über eine hohe Krankenhaus- und Intensivbettenkapazität. Das Statistische Bundesamt zählt rund 1900 Allgemein- und Fachkliniken unterschiedlicher Größe, die sich der ambulanten und vor allem stationären Behandlung und Pflege ihrer Patientinnen und Patienten widmen [15]. Das System ist jedoch primär auf zivile Regelversorgung ausgelegt und arbeitet bereits im Alltagsbetrieb häufig nahe der Auslastungsgrenze. Bereits während der COVID-19-Pandemie wurde deutlich, dass Personalengpässe und Materialabhängigkeiten strukturelle Schwächen darstellen [14].

Insgesamt gibt es laut Statistischem Bundesamt (Stand 27.11.2025) in Deutschland rund 473.000 Krankenhausbetten, die für eine Verletztenversorgung in Frage kommen. Allerdings haben sich nur 650 Kliniken in Deutschland zum Traumanetzwerk der DGU zusammengeschlossen. In diesen Kliniken findet die Hauptlast der Traumaversorgung statt. Diese Kliniken verfügen aktuell über ca. 33.000 traumatologisch ausgewiesene Betten. Zudem stehen 24.920 Intensivbetten (DIVI-Register November 2025) zur Verfügung, von denen etwa 9000 als High-Care-Intensivplätze gelten [15, 16]. Diese Kapazitäten erscheinen auf den ersten Blick hoch, müssen jedoch im Kontext der normalen Auslastung betrachtet werden [17]. Bereits im Routinebetrieb sind vielerorts die vorhandenen Kapazitäten ausgelastet, insbesondere in der Intensivmedizin sowie der spezialisierten Versorgung. Katastrophen-Krisen-Krieg(3K)-Szenarien, vor allem Kriege, stellen das Gesundheitssystem vor Herausforderungen, die weit über zivile Katastrophen hinausgehen. In einem Scoping Review kommen Wurmb et al. 2024 zur Kernaussage, dass Gesundheitssysteme überlastet sein können, und dass eine strukturierte Anpassung medizinischer Standards der vorherrschende Ansatz bei Katastrophen, Krisen und Kriegen ist. „Krisenversorgungsstandards stellen den etabliertesten Rahmen dar, obwohl es an international anerkannten Definitionen und Triage-Konzepten mangelt“ [18].

Große militärische Konflikte führen zu massenhaften Verletzungen durch Explosionen, Verbrennungen, Polytraumata und komplexen Amputationen [19]. Eine Fragebogenstudie von Thies et al. zur Vorsorge für Massenunfälle (TerrorMASCAL) in Bayern an 99 Kliniken ergab, dass die Kapazitäten der Notfallversorgung von Krankenhaus zu Krankenhaus stark variieren. Während überregionale Traumazentren für schwere Traumata und neurochirurgische Eingriffe gut ausgestattet sind, mangelt es kleineren regionalen und lokalen Zentren häufig an Personal und an dem für die Versorgung von Opfern extremer Gewalt erforderlichen spezialisierten chirurgischen Materialien [20]. Gleichzeitig kann die medizinische Infrastruktur selbst Ziel militärischer Angriffe werden. So hat die Weltgesundheitsorganisation (WHO) seit Beginn der Invasion 2022 in der Ukraine mehr als 2400 Angriffe auf medizinische Einrichtungen dokumentiert [36]. Diese Angriffe zeigen, dass Krankenhäuser und alle anderen mobilen und stationären medizinischen Einrichtungen in modernen Konflikten zunehmend selbst zum Ziel werden können.

Zudem hat sich die Krankenhauslandschaft in den vergangenen Jahrzehnten stark verändert. Seit den 1990er-Jahren ist sowohl die Zahl der Krankenhäuser als auch die Zahl der Krankenhausbetten deutlich zurückgegangen, während gleichzeitig die Zahl der behandelten Patienten gestiegen ist [16]. Diese Entwicklung führt zu einer steigenden Auslastung der vorhandenen Ressourcen und reduziert die verfügbaren Reservekapazitäten für außergewöhnliche Ereignisse wie Pandemien oder militärische Konflikte. Im internationalen Vergleich ergibt sich, dass deutsche Kliniken im Verteidigungsfall erheblich gefährdet sind, insbesondere bezüglich Energieversorgung, IT-Sicherheit, baulichem Schutz und organisatorischer Vorbereitung [2, 21–23]. Zudem zeigen internationale Umfragen, dass Ärzte in Deutschland häufig schlechter auf Katastrophenszenarien innerhalb und außerhalb von Krankenhäusern vorbereitet sind als ihre Kollegen weltweit [7, 8, 22]. Das neue Krankenhausversorgungsverbesserungsgesetz – KHVVG [24] könnte zu einer weiteren Reduktion von Betten und Intensivkapazitäten und damit der Verminderung der Resilienz führen, ohne dabei 3K-Szenarien zu berücksichtigen. Die Konzentrierung auf wenige Maximalversorger können unter Friedensbedingungen sinnvoll, im Verteidigungsfall aber kontraproduktiv sein.

Planungen der Bundeswehr gehen derzeit davon aus, dass bei einem Ausbruch von Kampfhandlungen auf dem Gebiet der NATO-Ostflanke mit bis zu 1000 verwundeten deutschen Soldaten pro Tag gerechnet werden muss [17, 25], die medizinische Versorgung benötigen. Hinzu käme die geflohene und evakuierte Zivilbevölkerung aus frontnahen Gebieten mit regulärem medizinischem Bedarf. Vertreter und Mitglieder des NATO-Militärbündnisses warnen, Moskau könne von 2029 an zu einem Angriff fähig sein. Zur Versorgung einer solchen Patientenzahl wären laut Planungen der Bundeswehr ca. 15.000 traumatologisch ausgewiesene Krankenhausbetten nötig [25], was ca. 45 % der aktuell ausgewiesenen traumatologischen Betten entspricht. Damit wird deutlich, dass auf weitere Krankenhausbetten anderer Zuordnung ein erheblicher Druck entstehen wird. Da zurzeit der Bundeswehr nur etwa 1800 Betten im eigenen Sanitätsdienst zur Verfügung stehen [26] und ein großer Teil des militärischen Personals sich in der Nähe des Kampfgeschehens befinden wird, müsste der weit überwiegende Teil der verwundeten Soldaten in zivilen Krankenhäusern versorgt werden, dies von postakuter Behandlung bis hin zu Rekonstruktion und Wiedereingliederung [25]. Das Gesundheitssystem der Ukraine zeigt, dass „die Mehrbelastung für ein ukrainisches Krankenhaus durch verwundete Soldaten bei bis zu 70 % gegenüber Friedenszeiten liegt,“ der Bedarf an Intensivkapazitäten habe sich verdoppelt, die Anzahl traumatologischer Patienten verdreifacht [27].

Bei extremer Ressourcenknappheit müssen verletzte Patienten triagiert werden. Zwißler et al. [28] kritisieren, dass gesetzliche oder normative Vorgaben, welche medizinisch begründete Priorisierungsentscheidungen einschränken oder widersprüchliche Handlungsanforderungen schaffen, das Erreichen des Ziels, möglichst viele Patienten zu retten, gefährden können. Insbesondere in 3K-Lagen bedarf es daher klarer, evidenzbasierter und praktisch umsetzbarer Triage-Konzepte, die den behandelnden Ärztinnen und Ärzten rechtliche Sicherheit bieten und gleichzeitig eine effiziente Nutzung knapper intensivmedizinischer Ressourcen ermöglichen. Andernfalls besteht die Gefahr, dass starre Regelungen zu einer faktischen Priorisierung nach dem Prinzip „first come, first served“ führen und dadurch die Gesamtzahl überlebender Patientinnen und Patienten sinkt.

Seit dem Angriffskrieg ab Februar 2022 durch Russland war das gesamte Gesundheitssystem der Ukraine entweder durch direkte Angriffe auf Kliniken oder durch fehlende Ressourcen oder Personalmangel betroffen. Problematisch ist für Krankenhäuser, aber auch für andere Gesundheitseinrichtungen der durch die Invasion verursachte akute Personalmangel, da viele Ärzte und Pflegekräfte entweder Binnenflüchtlinge wurden oder das Land verließen [29]. So liegen Berichte vor, dass Kliniken in umkämpften Landstrichen bis zu 30 % des Personals durch Weggang verloren, sodass teils fachfremdes Personal pflegerische, ärztliche oder technische Aufgaben übernehmen musste. Außerdem wurden bis 2023 in der Ukraine ca. 30.000 ärztliche und pflegerische Fachkräfte zum Armeedienst verpflichtet und standen somit dem zivilen Gesundheitssystem nicht mehr zur Verfügung [30].

Analysen der erwarteten Verletzungsmuster zeigen, dass etwa ein Drittel der Verletzten, die überwiegend im Kriegsgebiet versorgt werden, internistische oder allgemeinmedizinische Versorgung benötigt. Jedoch benötigen etwa 44 % chirurgische Eingriffe geringerer Komplexität und rund 22 % schwer chirurgische und intensivmedizinische Behandlung [2], wobei zu erwarten ist, dass alle zu operierenden deutschen Soldaten nach Deutschland verbracht werden [25], da im Kriegsgebiet voraussichtlich keine Möglichkeit einer längerfristigen Behandlung bestehen wird. Diese Verteilung entspricht typischen Mustern moderner Konflikte, in denen Explosionsverletzungen, Amputationen, Traumata und Verbrennungen dominieren [19]. Die Erfahrungen aus dem Russisch-Ukrainischer Krieg zeigen dabei eine deutliche Zunahme von Explosions- und Verbrennungsverletzungen durch Drohnen und Artillerie: „Mehr als 80 % der Verletzungen beträfen die Extremitäten. Meist komme es zu Mehrfachverletzungen von Knochen, Hautweichteilmantel, Nerven und Gefäßen. Tatsächlich sind im ukrainischen Gesundheitssystem plastisch-mikrochirurgische Kompetenzen zur Behandlung von Komplexverletzungen kaum vorhanden. Amputationen sind daher die Regel“ [27]. Ein kritischer Punkt ist dabei die Versorgung mit Blutprodukten. Unter Massenanfallbedingungen (MANV militärisch) ist dies ein in Deutschland strukturell schwach entwickelter Bereich. Weder die nationalen Reserven der Blutspendedienste noch die Military-Blood-Banking-Konzepte sind hierfür ausreichend [31, 32].

Viele dieser Verletzungen erfordern hochspezialisierte chirurgische Versorgung, intensive Therapie sowie lange Rehabilitation. Hinzu kommt, dass der Gesundheitsausschuss des Deutschen Bundestages diskutierte, dass die Standards der zivilen und militärischen Versorgung bisher weitgehend getrennt sind und zusammengeführt werden müssten, wenn im Ernstfall mehrere hundert Verletzte pro Tag versorgt werden sollen [21], wobei der Sanitätsdienst der Bundeswehr klar ausgedrückt hat, dass weiterhin planerisch verfolgt werde, dass Soldaten eine medizinische Versorgung zustehe, die im Ergebnis dem fachlichen Standard im Heimatland entspricht [25]. Diese zivil-militärische Zusammenarbeit würde eine enge Koordination zwischen Sanitätsdienst der Bundeswehr, Rettungsdiensten, zivilen Krankenhäusern und auch niedergelassenen Praxen erfordern. Ein weiterer kritischer Faktor ist die medizinische Evakuierung. Verwundete müssen häufig über große Entfernungen transportiert werden, bevor eine definitive Behandlung möglich ist. Entsprechende Konzepte wurden während der COVID-19-Pandemie mit dem sogenannten Kleeblatt-System zur Patientenverteilung entwickelt sowie teilweise erprobt [17], jedoch ist aufgrund der erheblichen Quantitätsdifferenzen zweifelhaft, ob diese auch bei der zu erwartenden um ein Vielfaches höheren Anzahl an Verletzten mit grundlegend anderen Verletzungsmustern tragfähig wären.

Zur Frage, inwieweit neben der Ukraine international Kliniken auf eine militärische Auseinandersetzung vorbereitet sind, gibt es überraschend wenig Literatur. Vergleichende Untersuchungen zwischen deutschen Kliniken und Kliniken in anderen Ländern lassen sich nicht finden. Auch Länder an der NATO-Ostflanke beleuchten mehr die Krankenhaus- und Gesundheitssystemresilienz sowie die Krisenvorsorge, die sich nur indirekt auf ein militärisches Konfliktszenario übertragen lassen. Finnische Autoren untersuchten 28 Kliniken mit der Fragestellung, ob frühere Katastrophen zu einer besseren Krankenhausvorbereitung führen würden [33]. Überraschenderweise schnitten Krankenhäuser mit realer Katastrophenerfahrung nicht besser ab als Krankenhäuser ohne solche Erfahrung. Die Autoren betonen, dass die Ergebnisse hohe Relevanz für die aktuelle Diskussion über die Vorbereitung europäischer Krankenhäuser auf militärische Konflikte haben, da reale Ereignisse allein keine Garantie für bessere Vorbereitung darstellen. Khorram-Manesh et al. untersuchten 2021 die Frage, wie militärische und zivile Gesundheitssysteme gemeinsam Krisen bewältigen können, wenn einzelne Systeme an ihre Grenzen stoßen [34]. Die Autoren schlussfolgern: Moderne Gesundheitskrisen (einschließlich Kriegsszenarien) erfordern eine enge zivil-militärische Integration. Weder das militärische noch das zivile Gesundheitssystem können allein die Versorgung einer großen Zahl von Verwundeten sicherstellen.

Im Krieg sind schwedische Kliniken Teil eines nationalen Verteidigungssystems (Total Defence). Sie müssen einen Massenanfall von Verletzten bewältigen können, werden im Kriegsfall funktional in ein hybrides militärisch-ziviles System eingebunden, sind jedoch stark abhängig von regionaler Planung und Koordination. Sie haben solide Strukturen, aber die praktische Resilienz sei sehr variabel [34]. Die baltischen Staaten setzten auf zwei Säulen, NATO-Präsenz und kollektive Verteidigung und Nationale Widerstandsfähigkeit [35]. Dabei werden Krankenhäuser als Teil der kritischen Infrastruktur betrachtet; die Gesundheitsversorgung ist ein wesentlicher Bestandteil der nationalen Resilienz. Die Aufrechterhaltung medizinischer Versorgung im Krisen- und Kriegsfall wird als Element der Gesamtverteidigung verstanden; zivile Einrichtungen müssen in nationale Verteidigungs- und Krisenpläne integriert werden [35]. Die baltischen Konzepte weisen somit deutliche Parallelen zur Diskussion über Resilienz deutscher Kliniken sowie deren Vorbereitung auf militärische Auseinandersetzungen auf.

### Lösungsansätze

Der Krieg in der Ukraine bietet wertvolle empirische Erkenntnisse über die Funktionsweise eines Gesundheitssystems bei militärischen Auseinandersetzungen. Krankenhäuser in Kriegsgebieten arbeiten unter extremem Druck. In einigen großen ukrainischen Kliniken wurden innerhalb weniger Jahre zehntausende kriegsbedingte intensivmedizinische Behandlungen durchgeführt [27]. Die Erfahrungen zeigen, dass eine kontinuierliche Versorgung nur durch internationale Unterstützung, medizinische Evakuierung und die Einrichtung modularer Krankenhäuser möglich war [37]. Zu den wichtigsten Anpassungsmaßnahmen gehörten somit eine Dezentralisierung der Versorgung, der Ausbau der Notfall- und Traumaversorgung, internationale medizinische Evakuierung und die Einrichtung mobiler medizinischer Einrichtungen. Letztendlich erfordert die Versorgung großer Verwundetenzahlen eine effiziente nationale sowie internationale Koordination der Krankenhauskapazitäten. Übertragen auf die Vorbereitung des deutschen Gesundheitssystems auf Szenarien militärischer Auseinandersetzungen an der NATO-Ostflanke sollten deshalb folgende Punkte Berücksichtigung finden:

1. Schaffung von strategischen Reserven in der operativen Therapie und Intensivmedizin, flexible Bettenstationen, modulare Notfallkliniken bzw. sanitätsdienstliche Einrichtungen. Die spezifischen Beiträge der Anästhesiologie – Damage Control Resuscitation, perioperatives Intensivmanagement, Airway Management bei einem Massenanfall von Verletzten, Teleintensivmedizin sowie Koordinationsfunktion in der Klinikeinsatzleitung – sind hier zu nennen [7, 17]. Während die Unfallchirurgie den operativen Schwerpunkt bildet, tragen anästhesiologisch-intensivmedizinische Strukturen entscheidend zur Systemsicherheit bei [17, 38]. Bei der COVID-19-Pandemie zeigte sich, dass Krankenhäuser in Deutschland kurzfristig Kapazitäten erweitern können, wenn entsprechende organisatorische Strukturen und Vorkehrungen existieren. Heller et al. haben hierzu einen Stufenplan mit zeitlichem Horizont vorgestellt [17]. Simulationen zur Krankenhausplanung zeigen, dass Szenariomodellierungen ein wichtiges Instrument zur Planung solcher Reservekapazitäten sind [20, 39–41].
2. Zivile Ärztinnen und Ärzte verfügen häufig nur über begrenzte Erfahrung mit typischen Verletzungsmustern aus militärischen Konflikten, da entsprechende Traumata im zivilen Versorgungsalltag selten auftreten und militärische Erfahrungen nicht systematisch in die zivile Medizin transferiert werden [42]. Daher ist der flächendeckende Ausbau von Fortbildungsmaßnahmen zu kriegstypischen Verletzungen unbedingt notwendig [19]. Das systematische Review von Suda et al. anhand von 25 Publikationen [43] sowie das Scoping Review von Sing et al. [5] mit 46 Studien zeigen, dass eine starke wissenschaftliche Evidenz für regelmäßige Schulungen und Übungen zur Verbesserung der Triage-Qualität sowie zur Überprüfung von Krankenhausnotfallplänen gegeben ist. Damit rücken Ausbildung, Training und organisatorische Vorbereitung als zentrale Elemente der Krankenhausresilienz bei potenziellen militärischen Konflikten in den Vordergrund [5, 43]. Hierzu entwickelten Seyghalani et al. [44] ein validiertes Modell zur Bewertung der organisatorischen Resilienz von Krankenhäusern in Krisen- und Katastrophenlagen. Auf Basis von einer Literaturrecherche, Expertenbefragungen und einer quantitativen Validierung mit 371 Führungskräften identifizieren die Autoren fünf zentrale Dimensionen der Krankenhausresilienz: Verwundbarkeit (Vulnerability), Vorbereitung (Preparedness), Unterstützungsmanagement (Support Management), Reaktions- und Anpassungsfähigkeit (Responsiveness and Adaptability) sowie Erholung nach der Katastrophe (Recovery). Diese Dimensionen umfassen insgesamt 36 Einzelkomponenten und bilden ein belastbares Rahmenmodell zur Bewertung und zur Verbesserung der organisatorischen Widerstandsfähigkeit von Krankenhäusern.
3. Ein entscheidender logistischer Faktor ist die zielgerichtete Verteilung großer Patientenzahlen über große Entfernungen [14, 17]. Moderne Logistik- und Verwundetentransportkonzepte umfassen einen strategischen Patiententransport zu Land, zur See und in der Luft sowie mobile Intensivtransportkapazitäten. Dieser wird in der Regel durch die jeweiligen Streitkräfte organisiert und durchgeführt, in Deutschland durch das Kommando Gesundheitsversorgung der Bundeswehr [17, 45]. Im Russisch-Ukrainischer Krieg wurden beispielsweise Hospitalzüge zur Evakuierung Verwundeter eingesetzt; ein Konzept, das auch in Deutschland wieder vermehrt diskutiert wird [46].
4. Personalreserven und Mobilisierung: Eine militärische Landes- oder Bündnisverteidigung würde mit einem erheblichen Mehrbedarf an Personal einhergehen [3]. Notwendig wären hierzu zivil-medizinische Reserveprogramme, die Reaktivierung ehemaliger Ärztinnen und Ärzte sowie von Pflegekräften, eine Integration von Rettungsdiensten und Katastrophenschutzstrukturen, die schnelle Mobilisierung verfügbarer medizinischer Fachkräfte sowie der Einbezug des medizinischen Nachwuchses (z. B. Medizinstudierende sowie Auszubildende in Gesundheitsberufen) [17]. Allgemeinverfügungen würden eine sofort vollziehbare rechtliche Grundlage für eine zentrale Krankenhauskoordination ermöglichen, auch mit Befugnissen organisatorische Weisungen [17, 38]. Auch ist zu diskutieren, inwieweit in solch einem Szenario das Arbeitszeitgesetz ausgesetzt werden sollte. Erfahrungen aus Israel zeigen, dass selbst gut vorbereitete Gesundheitssysteme durch Mass-Casualty-Ereignisse an ihre Grenzen gelangen können. Nach dem Angriff vom 07. Oktober 2023 musste beispielweise das Tel Aviv Medical Center unter extremen Bedingungen gleichzeitig eine große Zahl von Verletzten behandeln, während ein erheblicher Teil des medizinischen Personals zum Militärdienst eingezogen wurde [23]. Diese Erfahrungen verdeutlichen, dass Redundanz, flexible Organisationsstrukturen und kontinuierliche Vorbereitung entscheidend für die Resilienz von Krankenhäusern sind.
5. Zivil-militärische Zusammenarbeit (ZMZ): Eine zentrale Rolle spielt die Zusammenarbeit zwischen militärischen und zivilen Gesundheitsstrukturen [17]. Die zivil-militärische Zusammenarbeit wurde in den vergangenen Jahren zur sogenannten ZMV 4.0 weiterentwickelt, die auf Landes- und Bündnisverteidigung ausgerichtet ist [17]. Die Europäische Kommission fordert hierfür: „establish comprehensive civil-military preparedness arrangements, develop standards for civilian-military dual use planning and investment, organise regular EU exercises to promote comprehensive preparedness“ [47]. Analysen der Reaktion auf die COVID-19 Pandemie zeigen, dass die Verbindung militärischer Ressourcen und ziviler Bevölkerungsschutzstrukturen entscheidend für die Bewältigung großer Krisen ist [48]. Das Centre of Excellence der NATO betont: „Angesichts zunehmender globaler Instabilität und schwindenden Vertrauens in Institutionen ist die Zusammenarbeit zwischen militärischen und zivilen Akteuren wichtiger denn je“ [49]. Zu den wichtigsten Elementen gehören gemeinsame Trainingsprogramme, Koordinierung der Patientenverteilung, gemeinsame Lagezentren und interoperable Kommunikationssysteme [10].
6. Strukturreformen des deutschen Krankenhauswesens: Neben militärmedizinischen Maßnahmen erfordert die Vorbereitung auf eine militärische Auseinandersetzung auch strukturelle Reformen des Gesundheitssystems: stärkere Zentralisierung hochspezialisierter Traumazentren, regionale Versorgungsnetzwerke, strategische Reserven für Krisen- und Katastrophenszenarien sowie die Berücksichtigung der Krisenvorsorge in der Krankenhausfinanzierung [17]. Historische Erfahrungen zeigen, dass Gesundheitssysteme im Krieg auf temporäre Hilfskrankenhäuser und verkürzte Liegezeiten zurückgreifen mussten, um Kapazitätsengpässe zu bewältigen [3, 50].

### Empfehlungen

Die Bundesregierung formulierte im Januar 2025 in einer Gesetzesinitiative für das wenig später vom Deutschen Bundestag beschlossene „Zeitenwende-Artikelgesetz“: „Angesichts des völkerrechtswidrigen Angriffskriegs Russlands auf die Ukraine und der sich daraus ergebenden grundlegenden Änderung der Sicherheitslage in Europa soll die personelle Einsatzbereitschaft der Bundeswehr erhöht werden“ [51]. Diese „Zeitenwende“ markiert seit dem 24. Februar 2022 eine radikale Kehrtwende in der deutschen Sicherheits- und Verteidigungspolitik. Mit einem 100-Milliarden-Euro-Sondervermögen und massiv erhöhten jährlichen Budgets soll die Bundeswehr zur kriegstüchtigen, modernen Armee aufgerüstet werden. Auf der anderen Seite finden sich jedoch beispielsweise im Gesundheitsversorgungsstärkungsgesetz (GVSG) vom Februar 2025 [52] keine Hinweise zur Vorsorge der zivilen Gesundheitsversorgung für eine mögliche militärische Auseinandersetzung. Hier ist die Politik gefragt, entsprechende gesetzliche Maßnahmen zu ergreifen und Regelungsbedarfe zu identifizieren. Naturgemäß können, nachdem jahrzehntelang eine gewisse „Blindheit“ bezüglich einer militärischen Auseinandersetzung zu verzeichnen ist, schon aus finanzieller Sicht nicht alle notwendigen Maßnahmen zur Resilienzsteigerung unverzüglich umgesetzt werden. Im Sinne einer „Kaltstartfähigkeit“ [17] können jedoch kurz- und langfristige Maßnahmen definiert werden. Es wird hier deshalb von den Autoren als Expertenkonsens ein Stufenplan für die nächsten Jahre empfohlen:

1. Kurzfristig (möglichst sofort)
  Prüfung und Aktualisierung von Alarm- und Einsatzplänen [5, 18]
  Schulungen der Kliniken zur Verbesserung der fachlichen Kompetenz bezüglich Kriegsverwundungen [19]
  Stärkung der einrichtungsindividuellen Resilienz durch z. B. Aufbau und Wälzung größerer Lagerbestände für Verbrauchsmaterial [40, 53]
  Gemeinsame Übungen mit Bundeswehr und Katastrophenschutz [47]
  Stärkung der Psychosozialen Notfallversorgung (PSNV) in Kliniken und Bevölkerung [54]
  Aufbau klinikinterner sowie regionaler Lagezentren [47]
  Vernetzung mit anderen Akteuren des Gesundheitssystems und relevanter kritischer Infrastrukturen auf regionaler Ebene [10]
  Ertüchtigung von in den Krankenhäusern vorhandenen unterirdischen Flächen für improvisierte geschützte Behandlungsbereiche
  Schaffung der rechtlichen Voraussetzungen zur zentralen Krankenhausorganisation [17]
2. Mittelfristig (2 bis 5 Jahre)
  Ertüchtigung und Ausbau der Notstrom‑, Wasser- und Abwasserversorgung [10]
  Etablierung redundanter IT- und Kommunikationssysteme [10, 41]
  Vornahme baulicher Schutzmaßnahmen (Zugangssicherung etc.) [10].
3. Langfristig (5 bis 10 Jahre)
  Ausbau und Umsetzung gesetzlicher Vorgaben zur KRITIS-Resilienz [40]
  Integration der zivilen Gesundheitsversorgung in die nationale Verteidigungsplanung [47]
  Berücksichtigung gehärteter Klinikbereiche in der Neubauplanung

Einschränkend ist zu bemerken, dass die Evidenz zur Vorbereitung von Krankenhäusern auf militärische Auseinandersetzungen überwiegend auf Expertenkonsensus, Beobachtungsstudien, Simulationsübungen und narrativen Übersichtsarbeiten basiert. Randomisierte, kontrollierte und verblindete Studien konnten im untersuchten Zeitraum nicht identifiziert werden. Dies ist insbesondere auf die ethischen und praktischen Limitationen der Erforschung seltener Großschadens- und Kriegsszenarien zurückzuführen. Die Aussagekraft der verfügbaren Evidenz ist daher begrenzt und erlaubt überwiegend indirekte Schlussfolgerungen hinsichtlich der tatsächlichen Einsatzbereitschaft deutscher Kliniken.

Die zur Verfügung stehenden Zahlen zur Anzahl der Verwundeten, benötigter Bettenkapazität und Intensivplätzen in ukrainischen Kliniken sind nur sehr bedingt auf Deutschland übertragbar, da unterschiedliche geographische Bedingungen gegeben sind, unterschiedliche Krankenhausstrukturen bestehen sowie eine unterschiedliche NATO-Einbindung besteht.

## Schlussfolgerung

Der Krieg in der Ukraine sowie die Kriege und Konflikte im Nahen Osten haben die Bedeutung resilienter Gesundheitssysteme in militärischen Konflikten eindrucksvoll verdeutlicht. Die zivile Krankenhausversorgung wird im Falle eines militärischen Konflikts eine entscheidende Rolle in der medizinischen Versorgung spielen, die zivil-militärische Zusammenarbeit ist hierzu essenziell. Allerdings werden dem Gesundheitssystem bisher hierfür keinerlei finanziellen Mittel zur Verfügung gestellt. Das deutsche Krankenhauswesen ist zwar hochleistungsfähig, jedoch aktuell nicht für großflächige militärische Konflikte ausgelegt. Erfahrungen aus dem Russisch-Ukrainischen Krieg zeigen, dass moderne Kriege enorme Zahlen schwer verletzter Patienten erzeugen und das Gesundheitssystem rasch an seine Belastungsgrenzen bringen. Während kleinere Verwundetenströme bewältigbar wären, würde ein großflächiger militärischer Konflikt zusätzliche militärische, logistische und internationale Unterstützung erfordern. Herausforderungen liegen nicht nur in der Infrastruktur, sondern in der Verfügbarkeit von qualifiziertem Personal sowie von Intensivkapazitäten und in der logistischen Resilienz. Eine stärkere Integration ziviler Krankenhäuser in die nationale Sicherheits- und Verteidigungsplanung sowie regelmäßige Übungen und Investitionen in resilienzsteigernde Maßnahmen [18, 40] könnten entscheidend dazu beitragen, die medizinische Versorgung im Krisenfall sicherzustellen. Hingewiesen sei hier insbesondere auf die kürzlich in dieser Zeitschrift erschienene Publikation von Heller et al. zum Thema, „Anästhesiologie als integrative Querschnittsdisziplin der klinischen Resilienz – Was Kliniken jetzt für den Hochlastbetrieb vordenken müssen“ [17].

## Fazit für die Praxis

- *Ausbau der Notfall- und Traumaversorgung*: Internationale medizinische Evakuierung, mobile medizinische Einrichtungen, internationale sowie nationale Koordination der Krankenhauskapazitäten.
- *Strategische Reserven:* Reserven in der operativen Therapie und Intensivmedizin, flexible Bettenstationen, modulare Notfallkliniken.
- *Spezifische Beiträge der Anästhesiologie:* Damage Control Resuscitation, perioperatives Intensivmanagement, Airway Management beim Massenanfall von Verletzten, Teleintensivmedizin, Koordinationsfunktion in der Klinikeinsatzleitung.
- *Typische Verletzungsmuster aus militärischen Konflikten:* Flächendeckender Ausbau von Fortbildungsmaßnahmen zu kriegstypischen Verletzungen.
- *Personalreserven und Mobilisierung:* Zivil-medizinische Reserveprogramme, Reaktivierung ehemaliger Ärztinnen und Ärzte sowie von Pflegekräften, Integration von Rettungsdiensten und Katastrophenschutzstrukturen, schnelle Mobilisierung verfügbarer medizinischer Fachkräfte, Einbezug des medizinischen Nachwuchses.
- *Strukturreformen des deutschen Krankenhauswesens:* stärkere Zentralisierung hochspezialisierter Traumazentren, regionale Versorgungsnetzwerke, strategische Reserven für Krisen- und Katastrophenszenarien, Berücksichtigung der Krisenvorsorge in der Krankenhausfinanzierung.
- *Ertüchtigung der Kliniken: *Ausbau der Notstrom‑, Wasser- und Abwasserversorgung, Etablierung redundanter IT- und Kommunikationssysteme, bauliche Schutzmaßnahmen.
- *Zivil-militärische Zusammenarbeit: *Gemeinsame Übungen mit Bundeswehr und Katastrophenschutz, Koordination zwischen Sanitätsdienst der Bundeswehr, Rettungsdiensten und zivilen Krankenhäusern.
- *Rechtliche Maßnahmen: *Schaffung rechtlicher Voraussetzungen zur zentralen Krankenhausorganisation, Refinanzierung von Übungen, Materialbeschaffung, Vorratshaltung und Wälzung größerer Lagerbestände für Verbrauchsmaterial.

## Data Availability

Die in dieser Studie erhobenen Datensätze können auf begründete Anfrage beim Korrespondenzautor angefordert werden.
